# Germline defects of familial hemophagocytic lymphohistiocytosis-related genes presenting as adult-onset peripheral T-cell lymphoma

**DOI:** 10.3389/fimmu.2024.1365975

**Published:** 2024-02-09

**Authors:** Chong Wei, Danqing Zhao, Song Xue, Hao Cai, Congwei Jia, Daobin Zhou, Wei Zhang

**Affiliations:** ^1^ Department of Hematology, Peking Union Medical College Hospital, Chinese Academy of Medical Sciences & Peking Union Medical College, Beijing, China; ^2^ Department of Bone Marrow Transplant, Beijing Lu Daopei Hospital, Beijing, China; ^3^ Department of Pathology, Peking Union Medical College Hospital, Chinese Academy of Medical Sciences & Peking Union Medical College, Beijing, China

**Keywords:** peripheral T-cell lymphoma, familial hemophagocytic lymphohistiocytosis, next-generation sequencing, germline mutation, cytotoxic function

## Abstract

Germline mutations in genes involved in perforin-granzyme-mediated cytotoxicity such as *PRF1*, *UNC13D*, *STX11*, and *STXBP2* were known to cause familial hemophagocytic lymphohistiocytosis (FHL). In this study, we reported a unique group of 3 patients with germline mutations of *UNC13D* and *STX11* genes and presented as adult-onset peripheral T-cell lymphoma (PTCL) with cytotoxic T-cell phenotype and atypical lymphoma presentations. CD107a degranulation assay and NK-cell activity analysis demonstrated impaired cytotoxic function of the NK/T-cells of the patients with FHL-related mutations. Gene expression profile study revealed that up-regulated genes of the cytotoxic T-cells were enriched in autoimmune-related pathways. It was possible that impaired cytotoxic lymphocyte-mediated immune surveillance and autoantigen stimulation may both participate in PTCL oncogenesis. Germline defects of FLH-related genes may represent a novel predisposing factor for PTCLs.

## Introduction

Peripheral T-cell lymphomas (PTCLs) are a heterogeneous group of mature T-cell and natural killer (NK)-cell neoplasms characterized by poor prognosis and aggressive clinical behavior (1). PTCLs account for 25%–30% of non-Hodgkin’s lymphomas (NHLs) in China, a proportion significantly higher than in Western countries (2). Due to the rarity and limited geographic distribution of the disease, driver mutations and genetic predisposing factors of PTCLs are poorly defined.

Hemophagocytic lymphohistiocytosis (HLH) is a life‐threatening syndrome characterized by pathologic activation of macrophages and cytotoxic T cells. Germline mutations in genes involved in perforin-granzyme-mediated cytotoxicity such as *PRF1*, *UNC13D*, *STX11*, and *STXBP2* were known to induce familial HLH (FHL) (3). Patients with FHL was commonly deemed to follow an autosomal recessive mode of inheritance, indicating that patients with FHL harbor biallelic mutations in these genes. In contrast, individuals carrying monoallelic mutations of such genes may present atypically with later-onset HLH and relatively indolent course (4). Furthermore, recent studies have found that these FHL-related genes may increase the risk of lymphomas and Epstein-Barr Virus (EBV)-associated T/NK-cell lymphoproliferative disorders (5, 6). However, mutations of FHL related-genes in patients with PTCLs have been seldom reported and are still largely unknown.

Based on the emerging evidence that a certain relationship exists between genetic defects of FHL-related genes and lymphoma pathogenesis, targeted sequencing using a panel involving genes associated with hematological hereditary disorders and immunodeficiency disorders was performed on recently diagnosed PTCL patients in our center. Interestingly, germline defects of FHL-related genes were detected in 3 cases, who were diagnosed with PTCL of cytotoxic T-cell phenotype and presented with atypical clinical symptoms. In this study, we reported the clinical findings and genotypes of the 3 cases. More in-depth studies on the gene expression profile and cytotoxic function of the NK and T cells of the 3 cases were also reported.

## Patients and methods

### Patients and clinical data collection

This study included three patients diagnosed with PTCL, each harboring germline mutations in FHL-related genes. Baseline clinical characteristics, treatment modalities, and response assessments of the 3 cases were reviewed.

### Next-generation sequencing

Genomic DNA was extracted using the QIAamp DNA Blood Mini Kit (Qiagen, Hilden, Germany). Libraries were prepared using the KAPA HTP Library Preparation Kit (Kapa Biosystems, Boston, USA). For targeted enrichment, a customized panel of biotinylated oligoprobes (Roche NimbleGen) was designed to capture the coding regions of 700 genes associated with hematological hereditary disorders and immunodeficiency disorders. The captured DNA library was amplified and sequenced on Illumina Novoseq 6000 sequencer for paired reads at 150 bp.

### Isolation of CD8+ T-cells

Peripheral blood mononuclear cells (PBMCs) were isolated from peripheral blood samples using the Ficoll-Hypaque density centrifugation method. CD8+ T-cells were isolated from PBMCs via magnetic-activated cell sorting, using CD8 microbeads as per the manufacturer’s instructions (Miltenyi Biotech, Germany).

### RNA sequencing and data analysis

Total RNA was extracted from the suspension of isolated CD8+ T-cells by Trizol reagent (Invitrogen). The cDNA libraries were constructed using the VAHTS Universal V6 RNA-seq Library Prep Kit for Illumina (vazyme, Inc.). Clean reads were aligned to the human genome (GRCh38, Ensembl104) using the Hisat2 (7). EBSeq algorithm was applied to filter the differentially expressed genes (DEGs) under the following criteria: i) fold change (FC) > 1.5 or < 0.67; ii) false discovery rate (FDR) < 0.05 (8). Pathway annotation of DEGs was performed using the KEGG database. Fisher’s exact test was used to identify significant pathways with a threshold of significance defined by *P* < 0.05.

### CD107a degranulation assay and NK-cell activity analysis

CD107a degranulation assay and NK-cell activity were analyzed utilizing the flow cytometry method. For the CD107a degranulation assay, PBMCs isolated from peripheral blood samples of the patients and cultivated K562 cells were co-cultured at an effector-to-target ratio of 10:1. A basal degranulation group (PBMCs + culture medium) and a positive control group (PBMC + PMA and ionomycin containing medium) were also prepared. Cells were labeled with anti-human anti-CD107a-PE antibody. Anti-CD56-APC and anti-CD3-PerCP antibody were used to differentiate T- and NK-cells. After co-culture and staining, the samples were analyzed by flow cytometry. Surface expression of CD107a was assessed in the T- and NK-cells and compared before and after stimulation. Results are reported as ΔCD107a (percentage CD107a+ cells of stimulated – percentage CD107a+ cells of unstimulated sample) and measured by mean fluorescence intensity (MFI).

For the NK-cell activity test, the K562 target cell line with an enhanced stable expression fluorescent was established using the lentivirus-mediated transfection method. PBMCs of the patients and transfected K562 cells were co-cultured. The annexin V-PE/7AAD double staining marker was used for apoptosis labeling. After co-culture and staining, the samples were analyzed by flow cytometry. The percentage of apoptotic target cells reflected the cytotoxic activity of NK cells.

## Results

### Case presentation

**Figure 1:**
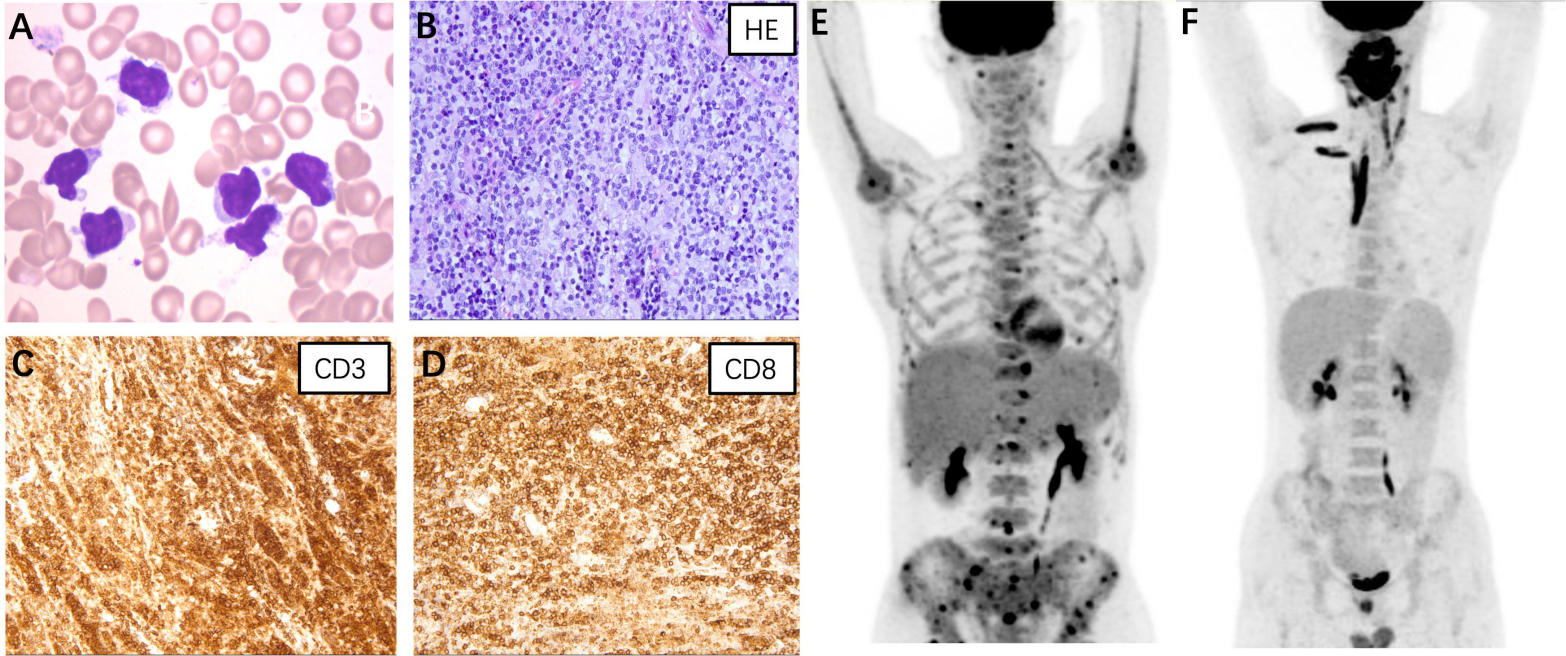
**(A)** Atypical lymphocytes were noticed on the bone marrow smear of case 1. The lymphoma cells were irregularly shaped, some with tails or pseudopodia, and some with a small amount of azurophil granules (Wright-Giemsa stain, ×1000). **(B–D)** Histopathological findings of case 3. **(B)** Hematoxylin and eosin staining revealed diffuse lymphocytes infiltrating in the salivary gland (×400); Immunostaining for CD3 **(C)** and CD8 **(D)** were positive (×400). Positron emission tomography/computed tomography of case 1 **(E)** and case 3 **(F)**.

**Case 1:** A 45-year-old male presented with a 12-year history of cytopenia and splenomegaly and 1 month of limb weakness. In 2010, leukopenia, thrombocytopenia, and splenomegaly were first recognized in a routine blood test without any symptoms. Atypical lymphocytes were noticed on bone marrow smear whereas a trephine biopsy showed no abnormalities. A splenectomy was performed in 2019 due to worsening fatigue and cytopenia. Pathological findings showed only splenic congestion without evidence of lymphoma. The patient’s symptoms were relieved and the blood cell count returned to normal after splenectomy. However, in 2021, the patient developed progressive limb numbness and weakness. He had no fever and tests for ferritin, triglyceride, and fibrinogen were within normal range. A repeated bone marrow assessment indicated an elevated proportion of lymphocytes (88%) with abnormal morphology (**Figure 1A**). Clonal rearrangement of the TCR Vβ gene was confirmed by PCR method. The diagnosis of T-cell lymphoma was confirmed by bone marrow biopsy. The immunophenotype of the T-lymphocytes was positive for CD2, CD3, CD5, CD8, and TCRαβ with weak expression of CD57 by flow cytometry. Positron emission tomography (PET)/computed tomography (CT) showed increased ^18^F-fluorodeoxyglucose (FDG) uptake in the lymph nodes, left cervical plexus, and bone marrow (**Figure 1E**). The patient was treated with six cycles of bendamustine and achieved complete remission. As of the latest follow-up in October 2023, the patient remains in remission.

**Case 2:** A 34-year-old male presented with a 2-year history of cytopenia and splenomegaly and a 3-month history of cervical lymphadenopathy. Leukopenia and splenomegaly were first noticed in 2021 without symptoms. He then developed cervical lymphadenopathy and multiple subcutaneous nodules with mild anemia and thrombocytopenia in 2022. The patient did not show fever during the clinical course and results for HLH-related tests (complete blood count, ferritin, triglyceride, and fibrinogen) were unremarkable. Bone marrow smear revealed an elevated proportion of lymphocytes with abnormal morphology. The abnormal T-lymphocytes were positive for CD3, CD7, CD8, and TCRαβ, with weak expression of CD5 and CD57 by flow cytometry. The patient was diagnosed with T-cell lymphoma following bone marrow biopsy. Further biopsies of the cervical lymph node and subcutaneous nodule were consistent with T-cell proliferative disease/lymphoma. PET/CT showed multiple FDG-avid lymph nodes in the neck, supraclavicular, right parotid gland, and subcutaneous nodules on the shoulders and back. The patient received 6 cycles of brentuximab vedotin combined with bendamustine and achieved partial remission. However, the disease progressed 5 months later with liver involvement. The patient was treated with thalidomide combined with cyclophosphamide and dexamethasone and was preparing for allogeneic hematopoietic stem cell transplantation (allo-HSCT).

**Case 3:** A 20-year-old male presented with a 1-year history of swelling of the tongue and a 3-month history of right maxillofacial mass. Since May 2022, the patient has experienced progressive swelling of the tongue with surface ulceration and movement restriction. Corticosteroid treatment was initially effective, but symptoms worsened after discontinuation. The patient subsequently developed swelling in the right maxillofacial region and a soft tissue mass was palpable. He did not experience fever and hepatosplenomegaly during the course and results for HLH-related tests were unremarkable. A biopsy of the lower lip confirmed the diagnosis of PTCL. Immunohistochemical staining of the tumor cells was positive for CD3, CD5, CD7, CD8, granzyme B, and TIA (**Figures 1B–D**). PET/CT revealed significant ^18^F-FDG uptake in the nasopharyngeal, cervical lymph nodes, and muscles of the neck, right shoulder, and right erector spinae (**Figure 1F**). The patient received two cycles of cyclophosphamide, doxorubicin, vincristine, and prednisone therapy but showed no improvement. The patient was now treated with etoposide combined with dexamethasone and was preparing for allo-HSCT.

### Genotype of the 3 cases

**Figure 2:**
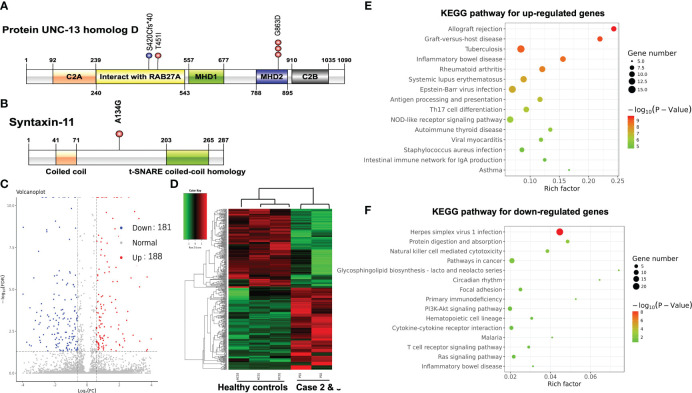
**(A)** Schematic locations of mutations in *UNC13D* gene product UNC-13 homolog D. **(B)** Schematic locations of mutations in *STX11* gene product Syntaxin-11. Volcano plot **(C)** and hierarchical clustering heatmap **(D)** of DEGs between PTCL patients and healthy controls. The bubble chart of the top 15 significant KEGG pathways of the up-regulated DEGs **(E)** and the down-regulated DEGs **(F)**.

NGS performed on the blood sample of case 1 revealed homozygous mutation of *UNC13D* c.2588G>A:p.Gly863Asp. NGS was also performed on blood samples of both parents of case 1 using the same panel. Both parents were determined as heterozygous carriers of *UNC13D* c.2588G>A mutation which confirmed the germline origin of this mutation. NGS performed on the bone marrow sample of case 2 identified compound heterozygous mutations of *UNC13D* c.2588G>A and *UNC13D* c.1259_1260 del:p.S420Cfs*40 with a variant allele frequency of around 50%. Genotyping of the buccal mucosa further confirmed the germline origin of these 2 mutations. NGS performed on the blood sample of case 3 identified two heterozygous mutations of *UNC13D* c.1352C>T: p.Thr451IlE and *STX11* c.401C>G: p.Ala134Gly. Germline origin of these two mutations was confirmed using the same method as case 1 Schematic locations of the mutations in *UNC13D* and *STX11* gene products of the 3 cases are depicted in **Figures 2A, B**.

### ACMG interpretation and in silico analysis

The impact of germline mutation in the 3 cases was evaluated according to the American College of Medical Genetics and Genomics (ACMG) guideline (9). As shown in **Table 1**, variant *UNC13D* c.2588G>A*, UNC13D* c.1352C>T, and *STX11* c.401C>G were all classified as uncertain significance according to the ACMG guideline. According to the gnomAD database, the frequencies of variant *UNC13D* c.2588 G>A and *STX11* c.401C>G were much higher in the East Asian population than in the European population (0.54% and 0.06% versus 0% and 0%). In silico analysis suggests that the amino acid position of *UNC13D* p.G863D and *UNC13D* p.T451I were predicted to be damaging by the SIFT (Sorting Intolerant From Tolerant) sorting algorithm. Variant *UNC13D* c.1259_1260 del is novel which resulted in a frameshift mutation starting at codon 420 and leading to a premature stop codon at the region that interacted with Rab27a. It is classified as likely pathogenic for FHL type 3 according to the ACMG guideline.

**Table 1:** Variant details and clinical significance.

Patient	Mutation type	Gene name	Nucleotide	Amino acid	Associated condition	ACMG interpretation	Clinvar	AF in East Asian*	SIFT prediction
Case 1	*UNC13D* homozygous mutation	*UNC13D*	c.2588G>A	p.Gly863Asp	FHL type 3	Uncertain significance	Reported	0.0054	Damaging
Case 2	*UNC13D* compound heterozygous mutations	*UNC13D*	c.2588G>A	p.Gly863Asp	FHL type 3	Uncertain significance	Reported	0.0054	Damaging
			c.1259_1260del	p.S420Cfs*40		Likely pathogenic	Not reported	N/A	N/A
Case 3	*UNC13D* and *STX11* heterozygous mutations	*UNC13D*	c.1352C>T	p.Thr451IlE	FHL type 3	Uncertain significance	Not reported	0.0000	Damaging
		*STX11*	c.401C>G	p.Ala134Gly	FHL type 4	Uncertain significance	Not reported	0.0006	Tolerated

ACMG, American College of Medical Genetics and Genomics; FHL, familial hemophagocytic lymphohistiocytosis; AF, allele frequency; SIFT, sorting intolerant from tolerant.

*according to gnomAD database (https://www.internationalgenome.org/).

### Gene expression profile study and cytotoxic function analysis

**Figure 3:**
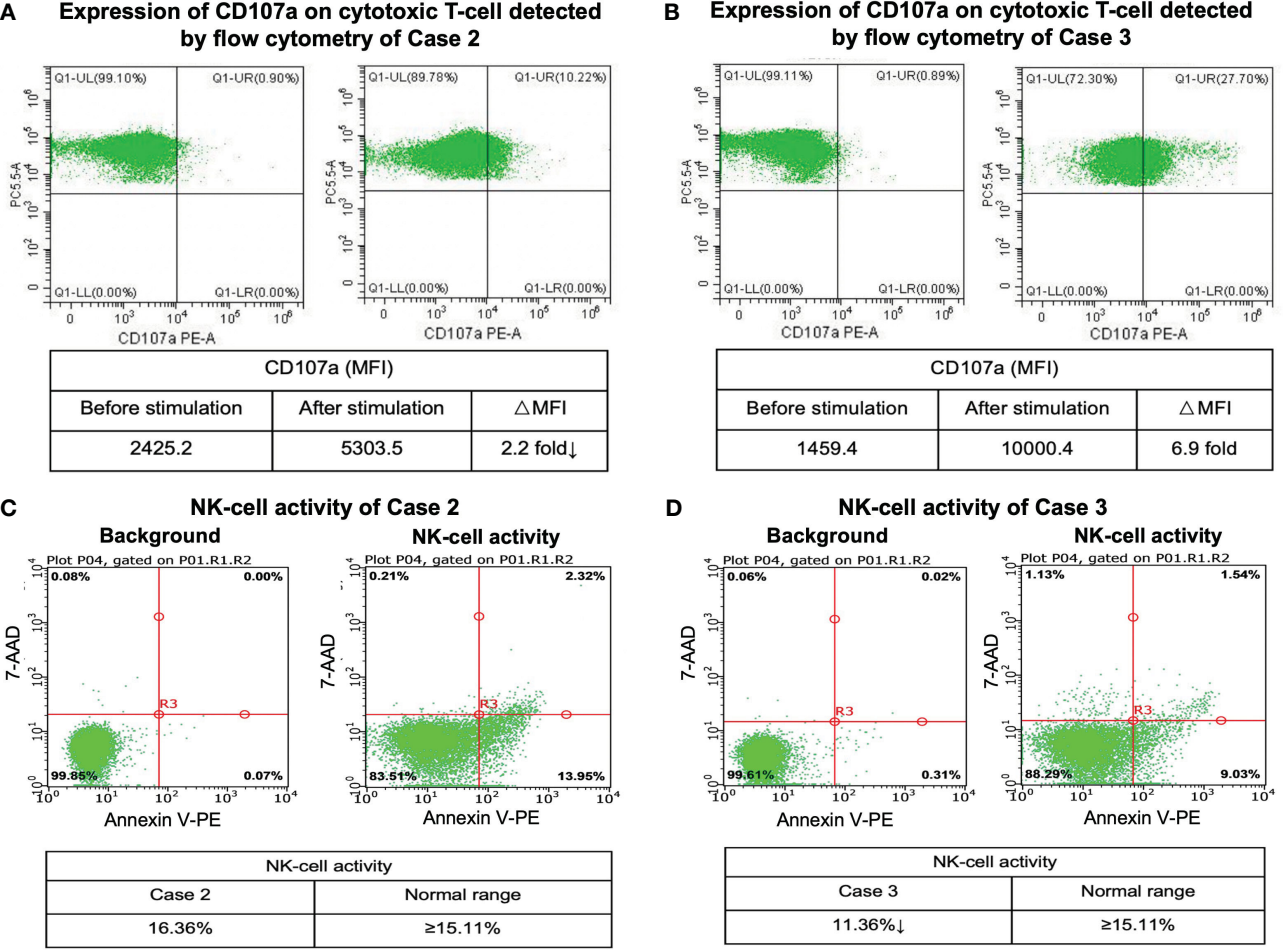
CD107a degranulation assay and NK-cell activity results. Flow cytometry figures represent the surface expression of CD107a on the cytotoxic T-cells before and after stimulation of case 2 **(A)** and case 3 **(B)**. Flow cytometry analysis of NK-cell activity of case 2 **(C)** and case 3 **(D)**.

We further compared the gene expression profile of the peripheral blood cytotoxic T-cells between PTCL group (case 2 and case 3), and 3 healthy controls. A total of 369 DEGs were identified between the PTCL group and healthy controls, including 188 up-regulated and 181 down-regulated genes (**Figures 2C, D**). For pathway analyses, the up-regulated DEGs were mainly enriched in autoimmune disease-related pathways and the down-regulated DEGs were mainly enriched in NK-cell mediated cytotoxicity and T-cell receptor signaling pathway. The top 15 significant KEGG pathways are shown in **Figures 2E, F**. For cytotoxic function analysis, CD107a degranulation assay and NK-cell activity were performed utilizing flow cytometry analysis (**Figure 3**). Following stimulation with K562 cells, the mean fluorescence intensity (MFI) of CD107a of cytotoxic T-cells of case 2 increased only 2.2 fold, indicating impaired cytotoxic activity (the normal range reference was defined as  > 2.8 fold). The CD107a degranulation assay result of case 3 was within the normal range. NK-cell activity of case 3 was decease, while that of case 2 was within the normal range.

## Discussion

Individuals with germline homogeneous mutations in genes essential for lymphocyte cytotoxicity, including PRF1, UNC13D, STX11, and STXBP2, typically present with familial hemophagocytic lymphohistiocytosis (FHL), a life-threatening immune dysregulation disorder characterized by marked hyperinflammation from uncontrolled activation of T-cells and macrophages. Deficiencies in these genes compromise cytolytic granule exocytosis at the immunological synapse which impairs the cytotoxic activity of T/NK-cells (10, 11). Homozygous mutations of *UNC13D* and *STX11* genes were responsible for FHL type 3 and type 4, respectively. Interestingly, the 3 cases with mutations of FHL-related genes presented as PTCL with cytotoxic T-cell immunophenotype in young adults rather than outbreak-fatal FHL during infancy. The lymphoma presentations of the 3 cases were also atypical which showed less aggressive clinical course and more extranodal involvements compared with typical PTCLs.

Recent studies have revealed a potential relationship between genetic defects of cytotoxicity and lymphoma predisposition. In the study conducted by Chen et al, variant *UNC13D* c.2588G>A was reported to be a founder mutation for lymphoma in the Chinese population (5). The allele frequency of *UNC13D* c.2588G>A in lymphoma patients was significantly higher compared with that in the healthy controls (10% versus 1.9%), suggesting that haploinsufficiency of this gene may predispose patients to lymphomas. Interestingly, in a recent case report, Yang T et al. described a case of PTCL complicated by HLH, in which germline heterozygous mutations of *UNC13D* and *CD27* genes were detected (12). However, all 3 patients in our study did not show symptoms of HLH before the diagnosis and during the clinical course of PTCL. Moreover, in the study conducted by Guan et al, germline mutations in *UNC13D*, *LYST*, and *PRF1* were reported to be linked to EBV-associated T/NK-cell lymphoproliferative diseases in Chinese patients (6).

The pathogenesis of PTCL on the basis of germline FHL-related gene mutations remains elusive. Based on the findings of our study, the cytotoxic function of NK-cell and T-cell is impaired as demonstrated by CD107a degranulation assay and NK-cell activity analysis. The impaired cytotoxic function of NK-cell and T-cell may further compromise the immune surveillance which predisposes an individual to lymphoma. In our study, up-regulated DEGs of the cytotoxic T-cells were significantly enriched in autoimmune disease-related pathways. It is possible that the disease started at an immune response to a persistent autoantigen, with clonal selection, expansion, and finally establishment of a monoclonal population. In this regards, we postulated that impaired cytotoxic lymphocyte-mediated immune surveillance and autoantigen stimulation may both participate in PTCL oncogenesis. The observation from this study hopefully will prompt more thorough investigations on the relationship between germline defects of cytotoxic lymphocytes and lymphoma pathogenesis.

In summary, we reported a unique group of 3 patients with germline defects of FHL-related genes and presented as PTCL with cytotoxic T-cell phenotype and atypical lymphoma presentation. Germline defects of FLH-related genes may represent as a predisposing factor for PTCLs. Further studies are warranted to understand the impacts of germline cytotoxic lymphocyte defects on the pathogenesis of PTCLs.

## Data availability statement

The original contributions presented in the study are included in the article/supplementary materials, further inquiries can be directed to the corresponding author.

## Ethics statement

The studies involving humans were approved by Peking Union Medical College Hospital. The studies were conducted in accordance with the local legislation and institutional requirements. The participants provided their written informed consent to participate in this study. Written informed consent was obtained from the individual(s) for the publication of any potentially identifiable images or data included in this article.

## Author contributions

CW: Conceptualization, Data curation, Formal analysis, Funding acquisition, Investigation, Methodology, Project administration, Resources, Software, Supervision, Validation, Visualization, Writing – original draft, Writing – review & editing. DQZ: Resources, Writing – original draft. SX: Resources, Writing – original draft. HC: Data curation, Writing – original draft. CJ: Data curation, Resources, Writing – original draft. DBZ: Resources, Writing – review & editing. WZ: Writing – review & editing.

## References

[1] Swerdlow SH Campo E Pileri SA Harris NL Stein H Siebert R. The 2016 revision of the World Health Organization classification of lymphoid neoplasms. Blood (2016) 127(20):2375–90. doi: 10.1182/blood-2016-01-643569 PMC4874220 26980727

[2] Liu W Ji X Song Y Wang X Zheng W Lin N. Improving survival of 3760 patients with lymphoma: Experience of an academic center over two decades. Cancer Med (2020) 9(11):3765–74. doi: 10.1002/cam4.3037 PMC7286476 32281275

[3] Canna SW Marsh RA. Pediatric hemophagocytic lymphohistiocytosis. Blood (2020) 135(16):1332–43. doi: 10.1182/blood.2019000936 PMC8212354 32107531

[4] Zhang K Jordan MB Marsh RA Johnson JA Kissell D Meller J. Hypomorphic mutations in PRF1, MUNC13-4, and STXBP2 are associated with adult-onset familial HLH. Blood (2011) 118(22):5794–8. doi: 10.1182/blood-2011-07-370148 PMC3228496 21881043

[5] Chen X Zhang Y Wang F Wang M Teng W Lin Y. Germline cytotoxic lymphocytes defective mutations in Chinese patients with lymphoma. Oncol Lett (2017) 14(5):5249–56. doi: 10.3892/ol.2017.6898 PMC5656022 29113160

[6] Guan YQ Shen KF Yang L Cai H Zhang M Wang J. Inherited genetic susceptibility to nonimmunosuppressed Epstein-Barr virus-associated T/NK-cell lymphoproliferative diseases in Chinese patients. Curr Med Sci (2021) 41(3):482–90. doi: 10.1007/s11596-021-2375-5 34170459

[7] Kim D Langmead B Salzberg SL. HISAT: a fast spliced aligner with low memory requirements. Nat Methods (2015) 12(4):357–60. doi: 10.1038/nmeth.3317 PMC4655817 25751142

[8] Leng N Dawson JA Thomson JA Ruotti V Rissman AI Smit BMG. EBSeq: an empirical Bayes hierarchical model for inference in RNA-seq experiments. Bioinformatics (2013) 29(8):1035–43. doi: 10.1093/bioinformatics/btt087 PMC3624807 23428641

[9] Richards S Aziz N Bale S Bick D Das S Gastier-Foster J. Standards and guidelines for the interpretation of sequence variants: a joint consensus recommendation of the American College of Medical Genetics and Genomics and the Association for Molecular Pathology. Genet Med (2015) 17(5):405–24. doi: 10.1038/gim.2015.30 PMC4544753 25741868

[10] Menager MM Menasche G Romao M Knapnougel P Ho C-H Garfa M. Secretory cytotoxic granule maturation and exocytosis require the effector protein hMunc13-4. Nat Immunol (2007) 8:257–67. doi: 10.1038/ni1431 17237785

[11] Neeft M Wieffer M de Jong AS Negroiu G Metz CHG van Loon A. Munc13-4 is an effector of rab27a and controls secretion of lysosomes in hematopoietic cells. Mol Biol Cell (2005) 16:731–41. doi: 10.1091/mbc.e04-10-0923 PMC545907 15548590

[12] Yang T Chen R Zhang M Jing R Geng J Wei G. Relapsed/refractory peripheral T-cell lymphoma-associated hemophagocytic lymphohistiocytosis with UNC13D and CD27 germline mutations. Cell Transplant. (2024) 33:9636897231221887. doi: 10.1177/09636897231221887 38183241 PMC10771736

